# Taste disorders and alopecia in myasthenia gravis

**DOI:** 10.1186/s12883-024-03644-w

**Published:** 2024-04-25

**Authors:** Akiyuki Uzawa, Shigeaki Suzuki, Satoshi Kuwabara, Hiroyuki Akamine, Yosuke Onishi, Manato Yasuda, Yukiko Ozawa, Naoki Kawaguchi, Tomoya Kubota, Masanori P. Takahashi, Yasushi Suzuki, Genya Watanabe, Takashi Kimura, Takamichi Sugimoto, Makoto Samukawa, Naoya Minami, Masayuki Masuda, Shingo Konno, Yuriko Nagane, Kimiaki Utsugisawa

**Affiliations:** 1 Department of Neurology, Graduate School of Medicine, Chiba University, 1-8-1, Inohana, Chuo-ku, Chiba-shi, Chiba, 260-8670 Japan; 2 Department of Neurology, Keio University School of Medicine, Tokyo, Japan; 3 Department of Neurology, Neurology Chiba Clinic, Chiba, Japan; 4 Department of Clinical Laboratory and Biomedical Sciences, Division of Health Sciences, Osaka University Graduate School of Medicine, Osaka, Japan; 5 Department of Neurology, National Hospital Organization Sendai Medical Center, Sendai, Japan; 6 Department of Neurology, Hyogo Medical University, Nishinomiya, Japan; 7 Department of Clinical Neuroscience and Therapeutics, Hiroshima University, Hiroshima, Japan; 8 Department of Neurology, Kindai University Faculty of Medicine, Osakasayama, Japan; 9 Department of Neurology, National Hospital Organization Hokkaido Medical Center, Sapporo, Japan; 10 Department of Neurology, Tokyo Medical University, Tokyo, Japan; 11 Department of Neurology, Toho University Ohashi Medical Center, Tokyo, Japan; 12 Department of Neurology, Hanamaki General Hospital, Hanamaki, Japan

**Keywords:** Alopecia, Non-motor symptoms, Refractory, Taste disorders, Thymoma

## Abstract

**Background:**

Non-motor symptoms in myasthenia gravis (MG) are rarely confirmed. Although there are some small cohort studies, a large-systemic survey has not yet been performed.

**Methods:**

We investigated the incidence and clinical characteristics of patients with MG who had taste disorders and alopecia using data of 1710 patients with MG enrolled in the Japan MG Registry 2021.

**Results:**

Among them, 104 (6.1%) out of 1692 patients and 138 (8.2%) out of 1688 patients had histories of taste disorders and alopecia, respectively. Among the patients with MG, taste disorders were significantly more common in women, those with severe symptoms, refractory MG, or thymoma-associated MG, and were less common in those with ocular MG. The taste disorders often occurred after the onset of MG and often responded to MG treatments. Alopecia was more common in MG patients with a history of bulbar palsy and thymoma, and it often occurred before the onset of MG and sometimes responded to MG treatments. Multivariate logistic regression analysis revealed taste disturbance was associated with worst quantitative MG score and thymoma-associated MG; and alopecia was associated with thymoma-associated MG.

**Conclusion:**

Clinicians should be aware of the non-motor symptoms in MG, especially in patients with severe myasthenic symptoms and thymoma-associated MG.

## Introduction

Myasthenia gravis (MG) is an autoimmune disease characterized by muscle weakness with fatigue and mediated by autoantibodies against acetylcholine receptor (AChR) or muscle-specific tyrosine kinase (MuSK) [1]. Non-motor symptoms, such as pure red cell aplasia, immunodeficiency, alopecia areata, neuromyotonia, limbic encephalitis, myocarditis, and taste disorders, are known complications during the course of MG [2]. These symptoms certainly affect the quality of life of patients with MG. Taste disorders (predominantly sweet taste loss) and alopecia areata are overlooked non-motor symptoms in MG, but they are occasionally observed in thymoma-associated MG [2–5]. Although the coexistence of MG and taste disorders or alopecia has been reported, the incidence has not been fully elucidated. In the present study, we aimed to investigate the incidence and clinical characteristics of patients with MG who had taste disorders and alopecia using a large cohort.

## Materials and methods

### Subjects

This study was conducted by the Japan MG Registry study 2021 group [6]. MG was diagnosed based on its characteristic clinical features and the positivity of AChR/MuSK antibody or neuromuscular junction dysfunction tests (eyelid easy fatigability test, ice pack test, edrophonium test, repetitive nerve stimulation test, and/or jitter increase on the single-fiber electromyography test). Altogether, 1710 consecutive patients with MG who visited the sites included in the Japan MG Registry 2021 between April and October of 2021 were enrolled in this study. Their clinical data, such as the age of onset, disease duration, sex, AChR/MuSK antibody positivity, past treatment regimens, presence of thymoma, disease status when at its worst, and current treatment and disease status, were evaluated. The survey of taste disorders and alopecia was performed using a very simple questionnaire in the present survey: (1) the history of taste disorders and/or alopecia (yes or no); (2) association between MG onset and the onset of taste disorders and/or alopecia (before or after MG onset); (3) association between the timing of thymectomy and onset of taste disorders and/or alopecia (before thymectomy, after thymectomy, or thymectomy was not performed); (4) response to immune-treatments for MG (yes or no). A patient with refractory MG is defined as someone whose symptoms cannot be sufficiently improved, even though receiving two or more immunosuppressive oral therapies or a combination of oral immunotherapies and repeated fast-acting treatment [7], or who cannot tolerate therapy for adequate control due to the side effects and/or treatment burden [6, 8].

This study was approved by the ethics committee of each neurological center (Institutional Ethical Review Board No. R3-1 at Hanamaki General Hospital, the primary investigating institute) and written informed consent was obtained from all study participants.

### Statistical analysis

The Mann–Whitney *U* and Fisher’s exact tests were used for comparing differences in unpaired continuous measures and categorical variables, respectively. If two or more factors showed significant correlations with non-motor symptoms, multivariate logistic regression analyses were used to identify independent factors. Statistical analyses were performed using JMP Pro version 16.0.0 (SAS Institute Inc., Cary, NC, USA).

## Results

### Clinical manifestations of MG patients with taste disorders

Information on the history of taste disorders was available in 1692 out of the 1710 patients. Taste disorders were confirmed in 104 (6.1%) of 1692 patients. The clinical features of the patients with or without taste disorders are summarized in Table 1. The taste disorders group was more likely to be women (*p* = 0.0036), had high quantitative MG scores at the worst stage of the disease (*p* < 0.0001), had a history of bulbar palsy (*p* = 0.0060) or myasthenic crisis (*p* = 0.0053), be refractory (*p* = 0.0026) or thymoma-associated MG (*p* < 0.0001), and less likely to be ocular MG (*p* = 0.0005) than the non-taste disorders group. Multivariate logistic regression analysis revealed taste disturbance was associated with only worst quantitative MG score (*p* = 0.0165) and thymoma-associated MG (*p* = 0.0053) among these factors. Other clinical manifestations were similar between the two groups. Taste disorders often occurred after the onset of MG (69.7%) and responded to MG treatments (60.4%). Improvement of taste disorders was not necessarily related to the that of MG symptoms. The frequency of taste disorders increased to 11.0% (42/383) when analyzing only the patients with thymoma-associated MG.

**Table 1:** Clinical features between MG patients with and without taste disorders

	Taste disorders group (*n* = 104, 6.1%)	Non-taste disorders group (*n* = 1588, 93.9%)	P value
Female ratio	74.0% (77/104)	59.4% (942/1585)	0.0036
Age at MG onset, y	47.9 ± 14.6	48.1 ± 18.7	0.5972
Disease duration of MG, y	10.2 ± 8.3	12.2 ± 11.3	0.1825
Worst quantitative MG score	16.5 ± 8.0	12.5 ± 6.7	< 0.0001
History of bulbar palsy	64.4% (67/104)	50.0% (786/1569)	0.0060
History of crisis	16.3% (17/104)	7.8% (123/1567)	0.0053
MM or better status, current status	49.0% (51/104)	58.1% (922/1588)	0.0818
Refractory MG	33.7% (35/104)	20.3% (310/1530)	0.0026
MG subtype			< 0.0001
Ocular MG	8.7% (9/104)	22.4% (354/1578)	0.0005
EOMG	20.2% (21/104)	21.3% (336/1578)	0.9015
LOMG	15.4% (16/104)	20.8% (328/1578)	0.2103
TAMG	40.4% (42/104)	21.6% (341/1578)	< 0.0001
MuSKMG	1.0% (1/104)	3.0% (48/1578)	0.3628
SNMG	14.4% (15/104)	10.8% (171/1578)	0.1655
Taste disorders			
Onset after MG onset	69.7% (69/99)	-	-
Response to MG treatments	60.4% (58/96)	-	-
Onset after thymectomy	54.7% (35/64)	-	-

Values are presented as the mean ± standard deviation unless otherwise indicated

MG, myasthenia gravis; EOMG, early-onset MG; LOMG, late-onset MG; TAMG, thymoma-associated MG; MuSKMG, MuSK antibody-positive MG; SNMG, seronegative MG; MM, minimal manifestations

### Clinical manifestations of MG patients with alopecia

The history of alopecia was available in 1688 out of the 1710 patients. Alopecia was confirmed in 138 (8.2%) out of 1688 patients. The clinical features of the patients with or without alopecia are summarized in Table 2. The alopecia group showed a higher frequency of patients with a history of bulbar palsy (*p* = 0.0412) and thymoma-associated MG (*p* = 0.0057) than the non-alopecia group. Multivariate logistic regression analysis revealed alopecia was associated with only thymoma-associated MG (*p* = 0.0076), but not with bulbar palsy. The other clinical manifestations were similar between the two groups. Alopecia often occurred before the onset of MG (63.2%) and occasionally responded to MG treatments (44.3%). Improvement of alopecia was not necessarily related to that of MG symptoms and was generally poor. The frequency of alopecia increased to 11.8% (45/382) when analyzing only the patients with thymoma-associated MG. There were 17 patients who developed both taste disorders and alopecia.

**Table 2:** Clinical features between MG patients with and without alopecia

	Alopecia group (*n* = 138, 8.2%)	Non-alopecia group (*n* = 1550, 91.8%)	P value
Female ratio	67.4% (93/138)	59.7% (924/1547)	0.0845
Age at MG onset, y	45.5 ± 17.2	48.3 ± 18.6	0.1078
Disease duration of MG, y	13.0 ± 10.5	12.0 ± 11.2	0.0565
Worst quantitative MG score	13.8 ± 7.4	12.6 ± 13.8	0.1061
History of bulbar palsy	59.4% (82/138)	50.3% (770/1531)	0.0412
History of crisis	8.0% (11/138)	8.4% (129/1529)	1.0000
MM or better status, current status	60.9% (84/138)	57.2% (886/1550)	0.4197
Refractory MG	23.5% (32/136)	20.9% (312/1494)	0.5099
MG subtype			0.0428
Ocular MG	15.9% (22/138)	22.1% (340/1540)	0.1050
EOMG	23.9% (33/138)	21.0% (323/1540)	0.4466
LOMG	16.7% (23/138)	20.8% (320/1540)	0.2722
TAMG	32.6% (45/138)	21.8% (337/1540)	0.0057
MuSKMG	1.4% (2/138)	3.1% (47/1540)	0.4273
SNMG	9.4% (13/138)	11.2% (173/1540)	0.5742
Alopecia			
Onset after MG onset	36.8% (49/133)	-	-
Response to MG treatments	44.3% (47/106)	-	-
Onset after thymectomy	35.3% (24/68)	-	-

Values are presented as the mean ± standard deviation unless otherwise indicated

MG, myasthenia gravis; EOMG, early-onset MG; LOMG, late-onset MG; TAMG, thymoma-associated MG; MuSKMG, MuSK antibody-positive MG; SNMG, seronegative MG; MM, minimal manifestations; MM, minimal manifestations

## Discussion

In the present study, we investigated the frequency and clinical characteristics of patients with MG who had taste disorders and alopecia using a large cohort data. We found that 6.1% of MG patients had a history of taste disorders, which were predominantly related to the female, severe cases, refractory cases, and thymoma-associated MG cases, and are less common in cases with ocular MG; and 8.2% of patients had a history of alopecia, which was common in patients with a history of bulbar palsy and thymoma. Multivariate logistic regression analysis revealed taste disturbance was associated with worst quantitative MG score and thymoma-associated MG; and alopecia was associated with thymoma-associated MG.

Taste disorders are non-motor symptoms in MG, causing deterioration of the patients’ quality of life. The frequency of taste disorders in the general population is reported to be 0.9–17.3% [9]. A previous multicenter cooperative study in Japan [3] revealed that 16 (4.3%) out of 371 MG patients had a history of taste disorders; of these, seven patients had taste disorders that were possibly related to the side effects of drugs or other diseases. Therefore, nine (2.4%) out of 371 patients were considered to develop taste disorders that are associated with MG, with mainly the sweet taste being selectively impaired. In that previous study, all patients had thymoma-associated MG (type B2 thymomas were the most common) and the taste disorders were aggravated according to the disease activity and improved by immunotherapy for MG. The prevalence of taste disorders in our study (6.1%) is somewhat high, as compared to that of a previous study [3]. Although the possibility that part of patients with taste disorders may have not been directly associated with MG cannot be completely ruled out, the high prevalence of taste disorders in cases with severe symptoms or refractory in our study suggested the relationship between taste disorders and disease activity of MG. In fact, taste disorders in MG were essentially changed according to the disease activity of MG (e.g. onset timing and responses to MG treatments), indicating that taste disorders and MG may share the common immune-mediated pathogenesis. In patients with severe symptoms or thymoma may have highly activated or unique immune mechanisms, but medicine-induced mechanisms by aggressive treatments cannot be ruled out as the cause of taste disorders [10]. Although the certain mechanisms are unknown, taste disorders are assumed to be driven by a CD4^+^ T-cell-mediated mechanism [2]. Additionally, a high prevalence of taste disorders in thymoma-associated MG cases indicates the possibility that thymoma is involved in the pathogenesis of taste disorders. Hence, a further study is required. As in the previous study [3], we confirmed that the taste disorders in MG tend to be more common in women. The taste disorders may be underdiagnosed and should be recognized as one of the important non-motor symptoms in MG.

Alopecia is known as another non-motor symptom in MG. Alopecia areata is characterized by a sudden and patchy loss of the scalp hair and is a T-cell mediated autoimmune disorder that targets hair follicles and affects nearly 2% of the general population during their lifetime [11, 12]. Immunohistochemical analyses of the skin revealed that lymphocytes (predominantly CD8^+^ T cells rather than CD4^+^ T cells) infiltrate into the peribulbar area [2]. Bulbar paralysis, myasthenic crisis, generalized type, and thymoma complications are dominant features found in MG patients with alopecia [4, 5]. Patients with MG have a certain risk for complicating other autoimmune diseases [13] and alopecia areata may be part of this spectrum. In addition, we should also consider other causes of alopecia including concomitant autoimmune diseases, atopic diseases, emotional or physical stress, and genetic factors [11]. In fact, alopecia in MG was not necessarily parallel with the disease activity of MG (often preceded by MG and thymectomy and does not respond well to MG treatment), same as previous papers [4, 5], suggesting that alopecia and MG may have independent pathogenesis. Previous studies have reported that alopecia areata was confirmed 3.0–3.7% of Japanese patients with MG [4, 5], whereas 0.5% of Caucasian patients with MG [14]. The difference on the prevalence may be derived from the differences in their immunogenetic backgrounds [4]. The prevalence of alopecia in our study (8.2%) is somewhat high as compared to those of previous studies [4, 5], although part of patients with alopecia may have not been directly associated with MG. The high prevalence of alopecia in patients with thymoma-associated MG suggests the possibility that thymoma is involved in the pathogenesis of alopecia. We should be aware that alopecia areata is a serious cosmetic problem that strongly deteriorates the quality of life among patients.

The proportion of patients with taste disorders and alopecia areata were much lower than that of patients without taste disorders and alopecia areata in this study, hence we performed Bayesian statistics to correct a possible over estimation of the results. As a result, taste disorders and alopecia were found to be more common in thymoma-associated MG, even after applying Bayesian statistics.

Several limitations of this study should be stated. Because this is a retrospective study, objective examinations for taste disorders or alopecia were not performed, and some data were missing (e.g. exclusion of other causes other than MG, relationship with disease activity of MG, and thymic pathology of thymoma). The frequency of taste disorders varies greatly depending on the report; therefore, it is difficult to determine whether taste disturbance is more common in MG than in general population. Non-motor symptoms of MG may not be necessarily direct complications of MG and may be mediated by the other causes, because the prevalence of taste disorders/alopecia in our study were somewhat higher than previous papers. Unfortunately, we could not collect the detailed data about the cause, severity, onset age, nature, or duration of taste disorders and alopecia. There may be a certain difference in detailed symptoms of taste disturbances and alopecia between patients with and without thymoma, which cannot be regrettably addressed in this study due to lack of detailed information This study does not have a concurrent or historical control group. Moreover, the data were collected from multiple centers in Japan; therefore, we could not determine whether racial differences exist. Prospective study using objective examinations for taste disorders and alopecia would be required to address these limitations.

## Conclusions

Taste disorders and alopecia might be overlooked in patients with MG. These non-motor symptoms were frequently confirmed, especially in severe or thymoma-associated MG cases, and may be induced by unrevealed immune-mediated mechanisms. Further studies should be performed to reveal the mechanisms of non-motor symptoms in MG.

## Data Availability

No datasets were generated or analysed during the current study.
